# A severe bullous cutaneous anthrax case

**DOI:** 10.1590/0037-8682-0080-2023

**Published:** 2023-06-02

**Authors:** Nurten Nur Aydin, Murat Aydin

**Affiliations:** 1 Erzurum Regional Training and Research Hospital, Infectious Diseases and Clinical Microbiology Department, Erzurum, Turkey.

A 57-year-old man presented with swelling and a painless wound on the left arm. He was engaged in animal husbandry, and 10 days prior to this presentation, he had slaughtered a sick animal. A black, 2-cm ulcerative lesion was observed on his left forearm. No pathogens were detected in the culture obtained from the lesion. A real-time polymerase chain reaction assay of the serum sample showed *Bacillus anthracis*. 

**FIGURE 1:**
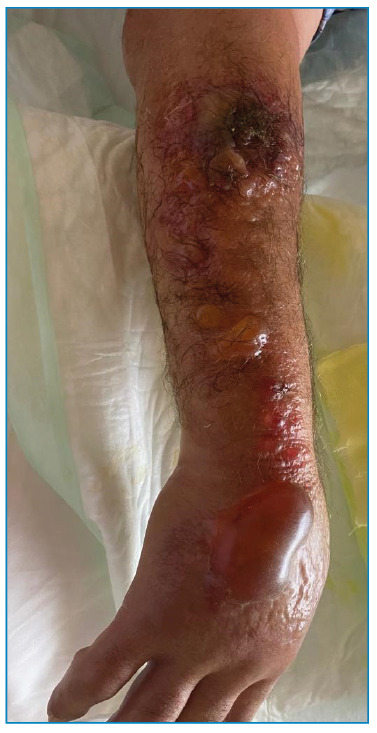
Multiple bullous lesions and edema seen on the third day of treatment.

**FIGURE 2:**
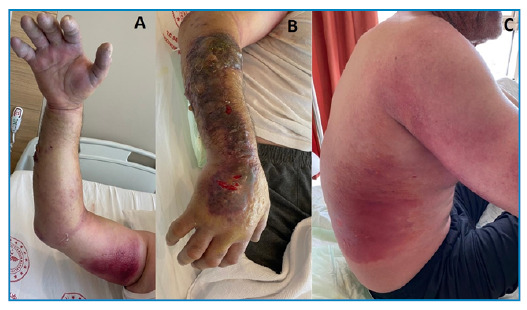
Appereance of cyanotic extremity **(A)**, edema and bullae spread to the entire arm **(B)**, and back **(C)**.

**FIGURE 3:**
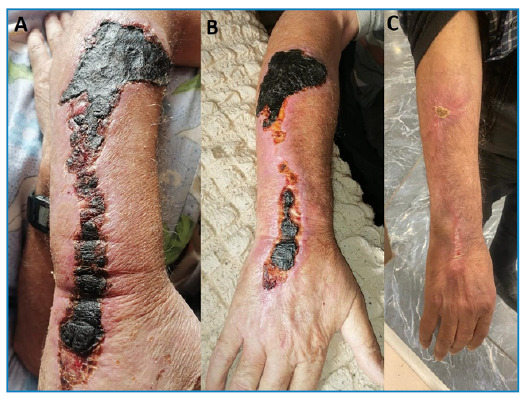
Appearance of the lesions at **(A)** 1 month, **(B)** 2 months, and **(C)** 3 months of follow-up.

Additionally, meropenem, linezolid, and ciprofloxacin were administered to him. On the 3rd day of treatment, bullae of various sizes developed around the lesion, and edema progressed significantly (Figure 1). Methylprednisolone 100 mg/day was included in his medications. The edema and bullae spread to his entire arm and the back (Figure 2). Treatment was completed within 21 days. The appearance of the lesions during follow-up is shown in Figure 3.

Anthrax is a zoonotic disease caused by *Bacillus anthracis,* a Gram-positive toxigenic endospore-forming bacillus. Its natural hosts are herbivores from which humans acquire diseases through incidental contact with infected animals or animal products^1^. This case demonstrates the importance of prompt diagnosis, timely initiation of appropriate antibiotic therapy, and supportive measures, including corticosteroids, in managing severe cutaneous anthrax. In some cases, steroids may control severe inflammation, particularly in patients with significant edema, involvement of the head and neck region, or anthrax meningitis^2^ ^,^ ^3^.

Further studies are warranted to elucidate the optimal management strategies for severe bullous cutaneous anthrax and guide clinical decision-making in similar cases.
